# Feedback Regulation of Pancreatic Juice Secretion in Pigs

**DOI:** 10.3390/biom16020322

**Published:** 2026-02-19

**Authors:** Jose Luis Valverde Piedra, Sylwia Edyta Szymanczyk

**Affiliations:** 1 Department of Pharmacology, Toxicology and Environmental Protection, Faculty of Veterinary Medicine, University of Life Sciences in Lublin, Akademicka 12, 20-033 Lublin, Poland; 2 Department of Animal Physiology, Faculty of Veterinary Medicine, University of Life Sciences in Lublin, Akademicka 12, 20-950 Lublin, Poland; sylwia.szymanczyk@up.edu.pl

**Keywords:** pancreatic secretion, negative feedback, bile acids, CCK, leptin, ileal brake, FXR/TGR5

## Abstract

Pancreatic exocrine secretion is regulated by the physicochemical properties and nutrient composition of gastric and intestinal chyme. The present study examined integrative feedback mechanisms involved in the physiological control of pancreatic secretion, with particular emphasis on interactions between pancreatic juice, bile, and gut-derived regulatory and metabolic signals. A chronic porcine model enabling selective withdrawal and controlled reintroduction of pancreatic juice and bile into defined intestinal segments was employed. Duodenal and ileal exposure to pancreatic juice suppressed pancreatic enzyme secretion, while intraduodenal administration of pancreatin elicited a biphasic inhibitory response. Interruption of bile flow to the duodenum resulted in increased pancreatic protein output and was associated with reduced circulating cholecystokinin concentrations. In contrast, intraduodenal infusion of bile acids attenuated pancreatic exocrine secretion. Prolonged bile deprivation led to sustained pancreatic hypersecretion accompanied by a marked reduction in biliary leptin output. Collectively, these findings indicate that pancreatic exocrine secretion in pigs is regulated by multiple interacting feedback pathways operating along the gastrointestinal tract. The observed responses support functional contributions of protease-dependent luminal feedback, distal intestinal sensing, hormone-dependent regulation, and bile-associated metabolic modulation.

## 1. Introduction

Exocrine pancreatic secretion is regulated by an integrated network of neural and hormonal mechanisms that adjust the delivery of digestive enzymes and bicarbonate to the composition and timing of gastric and intestinal chyme [1]. Although these regulatory pathways have been extensively studied, feedback mechanisms remain incompletely translated across species. This variability reflects differences in the relative contribution of CCK-dependent acinar stimulation, vagal reflex pathways, and ductal secretin signalling between rodents, large mammals, and humans [2].

The pig represents an established translational model for gastrointestinal physiology and nutritional research [3]. However, in this species, several aspects of pancreatic feedback regulation remain insufficiently resolved, particularly those related to bile components and distal intestinal sensing. One of the central elements of feedback control is protease-dependent modulation of CCK release. In humans, intraduodenal trypsin reduces circulating CCK concentrations, demonstrating that luminal proteolytic activity can down-regulate pancreatic enzyme secretion through hormonal pathways [4]. Rodent studies further identified trypsin-sensitive CCK-releasing peptides, including luminal CCK-releasing factor and monitor peptide, which accumulate when protease activity is reduced or functionally consumed by dietary substrates, thereby enhancing I-cell stimulation and pancreatic enzyme output [5,6,7]. Despite these advances, the range of luminal protease activity over which this feedback operates, as well as its saturation characteristics, are not well defined in pigs under chronic feeding conditions. It also remains unclear whether this regulation is restricted to the duodenum or extends to more distal intestinal segments. In large mammals, CCK-induced pancreatic secretion is often mediated predominantly through neural amplification, whereby CCK1 receptors on vagal afferents initiate vago-vagal reflexes that enhance cholinergic stimulation of acinar cells [2]. At the cellular level, CCK activates phospholipase C–dependent Ca^2+^ signalling in acinar tissue [8]. Importantly, porcine pancreatic tissue displays differences in CCK receptor expression and functional responsiveness compared with rodents, which complicates direct extrapolation of rodent-derived models [9]. In parallel, secretin remains the principal regulator of bicarbonate-rich ductal secretion via cAMP-dependent mechanisms [10]. Physiological pancreatic output therefore emerges from coordinated interactions between multiple regulatory pathways rather than from a single dominant secretagogue.

Beyond the classical protease–CCK axis, accumulating evidence indicates that bile and bile acids influence pancreatic exocrine secretion. However, the direction and magnitude of these effects vary considerably between experimental models. One contributing factor may be the highly dynamic nature of intestinal bile acid concentrations, which fluctuate markedly following gallbladder emptying and reach millimolar levels in the proximal intestine after feeding in humans, while systemic concentrations remain substantially lower [11,12]. These gradients enable bile acids to function as local intestinal signals capable of modulating enteroendocrine activity and neural reflexes rather than acting solely as detergents for lipid digestion. Two bile acid-responsive pathways are of particular interest in this context. Farnesoid X receptor (FXR) links intestinal bile acid exposure to endocrine feedback through FGF15/19 signalling [13], whereas the G protein–coupled receptor TGR5 (GPBAR1) increases intracellular cAMP and promotes incretin release, including GLP-1, thereby influencing gastrointestinal secretory regulation [14]. Although these pathways are well characterised in metabolic physiology, their integration with in vivo pancreatic enzyme feedback remains insufficiently examined, especially in large-animal models allowing independent manipulation of bile and pancreatic juice.

Importantly, intestinal feedback regulation may not be confined to the proximal small intestine. In pigs, luminal delivery of pancreatic juice or bile to different intestinal segments has been reported to modify pancreatic secretory output, suggesting that distal intestinal sensing mechanisms resembling an “ileal brake” may contribute to overall regulation [15,16].

This concept is physiologically plausible, as nutrient and bile acid detection in the ileum could modulate pancreatic secretion once digestion progresses distally, thereby limiting energetically inefficient enzyme oversupply [2]. Nevertheless, the relative contribution of bile acids and pancreatic enzymes to such distal feedback, as well as the hormonal mediators involved, remains unclear in weaned pigs studied under chronic cannulation conditions [17].

Finally, pancreatic exocrine secretion may also be influenced by metabolic hormones traditionally associated with appetite and energy homeostasis. Leptin has been reported to modulate pancreatic secretion and to interact with CCK-dependent pathways in a context- and dose-dependent manner in experimental models [18,19]. However, the potential role of biliary or intestinal leptin in phase-dependent fine-tuning of pancreatic exocrine output remains poorly characterised in large mammals. This limitation largely reflects the scarcity of studies combining direct measurements of bile composition with pancreatic secretory responses in conscious, chronically instrumented animals [17,20].

### Aim of the Study

**Figure 1:**
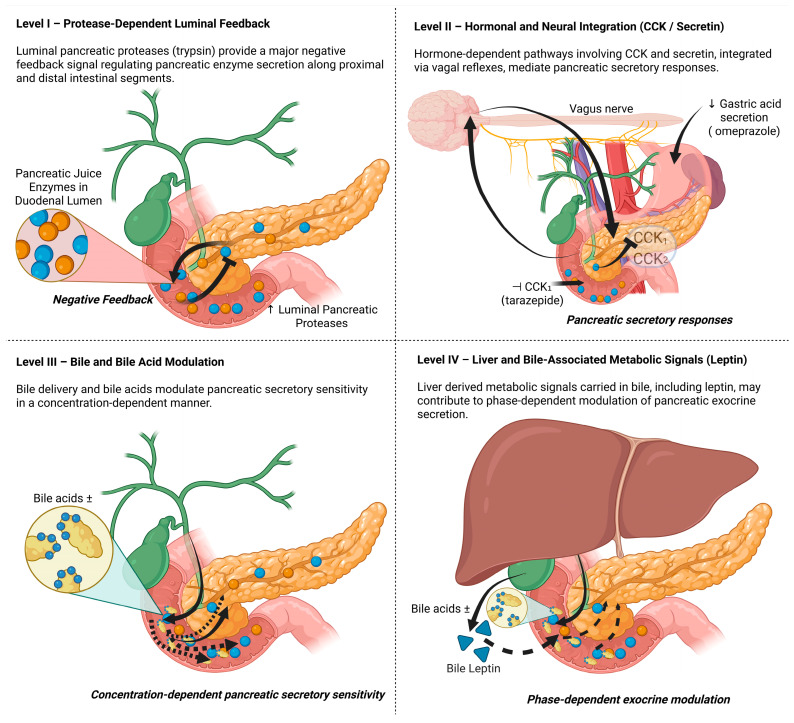
Conceptual framework of multilevel feedback regulation of pancreatic exocrine secretion in pigs. The scheme illustrates regulatory pathways investigated in the present study and does not imply exclusive or strictly sequential control. Created using BioRender (https://www.biorender.com/) [21].

The aim of this study was to investigate the relationships between four conceptual regulatory levels involved in pancreatic exocrine control (Figure 1). These levels comprised (i) luminal pancreatic juice and enzyme delivery as a primary protease-dependent feedback signal, (ii) hormone-dependent pathways with particular emphasis on cholecystokinin (CCK) and acid/secretin-dependent mechanisms, (iii) bile delivery and bile acid exposure as modulatory luminal and enteroendocrine signals, and (iv) bile-associated metabolic signalling, with specific focus on leptin as a potential phase-dependent modulatory factor. The study was conducted in weaned piglets equipped with chronic pancreatic and bile duct catheters and duodenal and ileal access.

In the present work, pancreatic exocrine regulation is conceptualised as a system of hierarchically organised feedback loops, defined as a multilevel control network in which pancreatic secretion is regulated sequentially and in parallel by direct luminal signals, hormone-dependent pathways, and bile- and metabolism-related modulatory mechanisms.

To investigate these relationships, two complementary chronic porcine experimental models were employed.

Using Experimental Model I, we tested the hypothesis that luminal delivery of pancreatic enzymes constitutes the primary inhibitory feedback mechanism controlling pancreatic exocrine secretion. Specifically, we hypothesised that graded intraluminal administration of pancreatic enzymes suppresses pancreatic secretion in a dose- and site-dependent manner along both proximal (duodenal) and distal (ileal) segments of the small intestine, including conditions of interrupted endogenous pancreatic juice flow.

Using Experimental Model II, we tested the hypothesis that bile delivery and bile acid exposure represent independent modulatory signals in the regulation of pancreatic exocrine secretion and that interruption of bile flow results in sustained stimulation of pancreatic enzyme output. This model further enabled evaluation of the contribution of CCK- and acid/secretin-dependent pathways through pharmacological blockade of CCK_1_ receptors and inhibition of gastric acid-stimulated secretin release, as well as assessment of bile-borne leptin as a potential metabolic signal associated with pancreatic secretory regulation.

By integrating selective withdrawal and controlled reintroduction of pancreatic juice and bile with targeted pharmacological interventions, this study aimed to delineate the relative contribution and functional hierarchy of luminal enzyme feedback, bile acid signalling, hormonal pathways, and bile-associated metabolic cues in the coordinated regulation of pancreatic exocrine function.

## 2. Materials and Methods

The study was designed to evaluate the regulatory mechanisms involved in feedback control of pancreatic exocrine secretion under controlled experimental conditions.

### 2.1. Animals

The experiments were performed on weaned piglets of a commercial crossbreed (Large White Swedish × Hampshire × Yorkshire) with an initial body mass of 15 ± 5 kg. Animals were obtained from the experimental farm in Odarslöv belonging to the Swedish University of Agricultural Sciences (Alnarp, Sweden).

All animal procedures were approved by the Local Ethics Committee of Lund University, Sweden (permission no. M238-99).

### 2.2. Preparation of Experimental Model I

#### 2.2.1. Surgical Procedures

The relationship between the activity of pancreatic proteolytic enzymes in the small intestine and pancreatic juice secretion was achieved using the model described by Pierzynowski et al., Thaela et al., and Rantzer et al. [15,22,23,24].

Piglets were premedicated with azaperone (2 mg/kg body weight; Stresnil, Janssen Pharmaceutica, Beerse, Belgium), and general anaesthesia was induced with halothane (3% in air; ISC Chemicals Ltd., Avonmouth, Bristol, UK). All surgical procedures were performed under aseptic conditions.

Animals were surgically fitted with a chronic pancreatic duct catheter and a T-shaped duodenal cannula made of medical-grade silicone, according to the original technique described by Pierzynowski et al. and subsequently modified by Thaela et al. [22,23]. A catheter was additionally placed in the external jugular vein to allow stress-free blood sampling during experimental procedures.

The pancreatic duct catheter enabled continuous collection of pancreatic juice, whereas the duodenal cannula allowed free return of pancreatic secretion to the intestinal lumen under control conditions. During experimental interventions, this cannula permitted controlled intraduodenal administration of pancreatic juice or test solutions. An additional catheter inserted into the ileum provided direct access to the distal small intestine for site-specific delivery of pancreatic enzymes.

#### 2.2.2. Postoperative Care and Animal Maintenance

After surgery, piglets were housed individually in pens with free access to drinking water. Visual contact between animals was maintained to minimise social isolation-related stress.

From the day of surgery until the end of the experimental period, animals were monitored daily. Clinical assessment included measurement of rectal temperature, heart rate, respiratory rate, general health status, and regular recording of body weight. Experimental procedures were initiated after a minimum recovery period of seven days, when piglets were clinically stable and exhibited normal feeding behaviour.

For five days following the surgical intervention, piglets received intramuscular antibiotic therapy. Postoperative analgesia was provided according to institutional veterinary recommendations, and animals were continuously monitored for signs of pain, infection, catheter-related complications, or reduced feed intake. Predefined humane endpoints included sustained weight loss, signs of systemic infection, persistent catheter occlusion, or behavioural indicators of distress.

To compensate for water and electrolyte losses resulting from temporary diversion of pancreatic juice from the duodenum, animals were provided with an oral mineral supplement (Salt Balance, Lantmännen, Stockholm, Sweden) throughout the recovery period and after each experimental procedure, in accordance with the manufacturer’s guidelines.

The established experimental models required continuous 24 h monitoring. Patency of catheters and duodenal cannulas was evaluated several times daily. These inspections were essential to prevent obstruction of pancreatic and biliary flow into the duodenum and to mitigate the risk of pancreatitis and cholecystitis. Particular attention was paid to the care of the skin surrounding the cervical catheter site, the abdominal integument, and the limbs and hooves, with the aim of reducing pruritus around healing wounds and minimising irritation caused by pancreatic enzymes. For this purpose, a topical emollient (Kylbalsam, ACO Hud AB, Stockholm, Sweden) and a protective cream against enzymatic irritation (Helosan, Pharmacia Animal Health AB, Stockholm, Sweden) were applied at least twice daily.

Animals were fed twice daily with a standard commercial pig feed mixture (Växfor, Lantmännen, Sweden; Table 1), administered in the morning (10:00–11:00) and afternoon (15:30–16:30). Feed portions (180–350 g), corresponding to approximately 1% of body mass, were consumed within one hour under both control and experimental conditions. Throughout the study, piglets had continuous access to drinking water.

#### 2.2.3. Experiments Using Model I

**Scheme A1:**
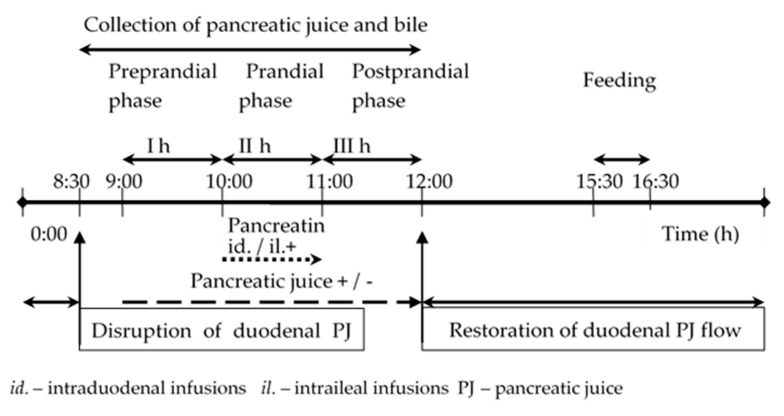
Exp. 1–14.

To evaluate the role of pancreatic juice in the small intestine, as well as the influence of the amount and site of pancreatic enzyme delivery on exocrine pancreatic function, a total of fourteen experimental protocols were performed using Model I. An overview of all experimental variants is provided in Appendix A.1 (Table A1), while the experimental design is illustrated in Appendix A.2 (Scheme A1).

In the control experiment (Experiment 1), pancreatic juice secretion was assessed during the perifeeding period under conditions of continuous return of pancreatic juice to the duodenum.

In Experiments 2–4, pancreatin solutions with different enzymatic activities (20%, 100%, and 200% of physiological trypsin activity) were infused intraduodenally while pancreatic juice flow to the duodenum was preserved.

In Experiment 5, pancreatic juice flow to the duodenum was completely interrupted.

In Experiments 6–8, pancreatin solutions with different enzymatic activities (20%, 100%, and 200%) were administered intraduodenally under conditions of eliminated pancreatic juice flow.

In Experiments 9–11, pancreatin solutions were infused into the ileum while pancreatic juice flow to the duodenum was preserved.

In Experiments 12–14, ileal pancreatin infusions were performed under conditions of disrupted pancreatic juice flow to the duodenum.

The complete experimental scheme is summarised in Table A1 (Appendix A.1).

#### 2.2.4. Collection of Pancreatic Juice

Experiments were conducted daily and lasted 3 h, comprising a 1 h pre-feeding period (preprandial phase), a 1 h feeding period (prandial phase), and a 1 h post-feeding period (postprandial phase).

Before the start of each experiment, the pancreatic catheter was disconnected from the duodenal cannula for 30 min. During this period, pancreatic juice was collected and subsequently reintroduced into the duodenum using a syringe in small aliquots during the first 30 min of the experimental session.

Pancreatic juice collection during experiments was performed under conditions allowing complete freedom of movement for the piglets. Each experimental phase was divided into two consecutive 30 min collection intervals. From each interval, a 0.5 mL sample of pancreatic juice was collected for subsequent biochemical analyses. The remaining volume was returned to the duodenum during the following 30 min in multiple small portions (2–6 mL), depending on the amount of secreted pancreatic juice.

#### 2.2.5. Pancreatin Infusions

Pancreatin infusions (Pancreatin, Solvay Pharmaceuticals GmbH, Hannover, Germany) were administered intraduodenally or intraileally starting immediately after the onset of feed intake and continued throughout the feeding (prandial) phase.

The reference trypsin activity defined as 100% corresponded to the mean enzymatic activity of pancreatic juice collected from piglets of the same age during the feeding phase under identical dietary conditions. Based on this reference, the pancreatin solution corresponding to 100% trypsin activity (3500 U trypsin) was prepared by dissolving 1.2 g of pancreatin in 24 mL of cooled physiological saline (0.9% NaCl).

Pancreatin solutions corresponding to 20% and 200% of physiological trypsin activity were prepared by dissolving 0.24 g and 2.4 g of pancreatin, respectively, in 24 mL of cooled physiological saline.

### 2.3. Preparation of Experimental Model II

To investigate the effect of the presence of bile in the small intestine on pancreatic juice secretion, the research model developed by Valverde Piedra and Pierzynowski, allowing for the collection and reintroduction of pancreatic juice or both pancreatic juice and bile simultaneously in the pig, was used [16].

#### 2.3.1. Experimental Design and Treatment Schedule

The study was conducted on six weaned piglets (*n* = 6) assigned to all treatments according to a Latin square, repeated-measures design.

Under control conditions (B+, PJ+), both pancreatic juice (PJ) and bile (B) were returned to the duodenum. In the experimental conditions, pancreatic juice was returned while bile was diverted (B−, PJ+), or both bile and pancreatic juice were diverted (B−, PJ−).

**Scheme A2:**
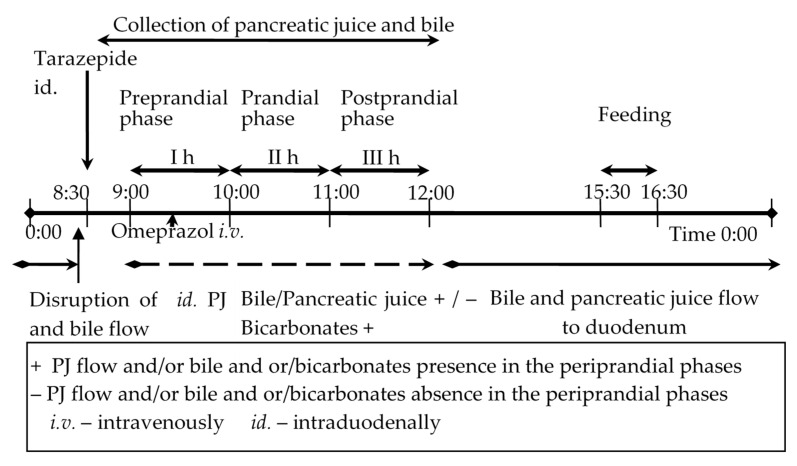
Exp. 15–21.

**Scheme A3:**
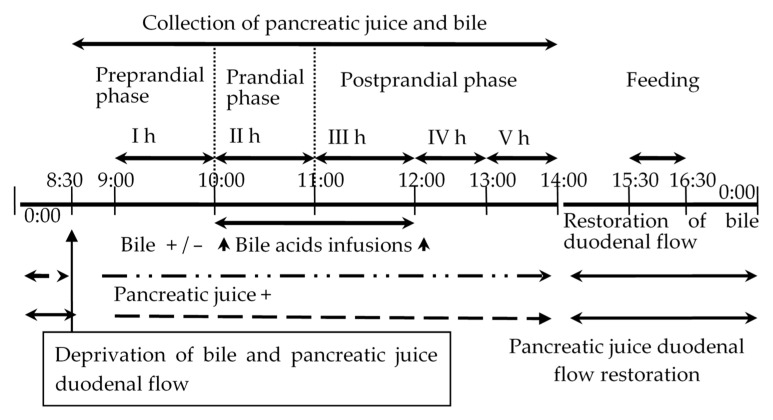
Exp. 22–26.

The experimental schemes for Experiments 15–21 and 22–26 are presented in Appendix A.1 (Table A2 and Table A3), and the corresponding protocol designs are illustrated in Appendix A.2 (Scheme A2 and Scheme A3).

Collection of pancreatic juice and bile was performed during the morning feeding session and lasted 5 h, comprising a 1 h preprandial period, a 1 h prandial period, and a 3 h postprandial period. Secretions were collected at 30 min intervals; volumes were recorded, and 1.5 mL aliquots were stored at −20 °C for subsequent analyses. The remaining pancreatic juice and bile were either reintroduced into the duodenum during the subsequent collection interval or chilled and reintroduced after completion of the experiments.

#### 2.3.2. Experiments Using Model II

To investigate the contribution of CCK1 receptor–mediated signalling and acid-dependent secretin release to feedback regulation of pancreatic exocrine secretion and bile output, experiments were performed using tarazepide (Solvay Pharmaceuticals GmbH, Hannover, Germany) omeprazole (Losec, AstraZeneca, Södertälje, Sweden), and bicarbonate for buffering of duodenal contents.

The experiments were conducted on six piglets surgically prepared according to Model II. Animals were additionally equipped with a catheter placed in the external jugular vein to allow intravenous administration of omeprazole. The experimental protocols followed the design outlined in Appendix A.1 (Table A2) and Appendix A.2 (Scheme A2).

Each experimental session lasted 3 h and included a preprandial, prandial, and postprandial phase. Body weight was measured before each experiment to calculate individual doses of omeprazole and tarazepide.

In control experiments (Experiment 15), pancreatic juice and bile secretion were measured under conditions of preserved return of both secretions to the duodenum. The effects of bile diversion alone (Experiment 16) and combined diversion of bile and pancreatic juice (Experiment 17) on pancreatic and biliary secretion were subsequently examined.

Pharmacological interventions were performed under conditions of preserved bile and pancreatic juice flow to the duodenum and included intravenous omeprazole administration (1 mg/kg body mass; Experiment 18), intraduodenal tarazepide administration (5 mg/kg body mass; Experiment 19), and combined administration of omeprazole and tarazepide at the same doses (Experiment 20).

In addition, pancreatic juice secretion was assessed under conditions of eliminated bile flow combined with intraduodenal bicarbonate infusion, administered in volumes corresponding to the bile output measured in individual collection intervals (Experiment 21). The doses of omeprazole and tarazepide were selected based on previously published studies [25,26,27].

#### 2.3.3. Collection of Pancreatic Juice and Bile

All experiments followed a 3 h experimental protocol, comprising a 1 h preprandial phase, a 1 h prandial phase, and a 1 h postprandial phase. Pancreatic juice and bile were collected in 30 min intervals. At the end of each collection interval, the volume of each secretion was measured, and a 1 mL aliquot was collected for subsequent biochemical analyses. In experiments involving the return of pancreatic juice and bile to the intestine, the collected secretions were reintroduced into the duodenum in small aliquots during the subsequent 30 min interval to mimic physiological flow patterns.

For pharmacological interventions, tarazepide was prepared *ex tempore* as a suspension in 5 mL of bile previously collected from the same piglet and administered intraduodenally at a dose of 5 mg/kg body mass, 90 min before the onset of feed intake. Omeprazole was administered intravenously at a dose of 1.0 mg/kg body mass, 30 min before feeding. The detailed experimental timeline and intervention sequence are illustrated in Appendix A.2 (Scheme A2).

#### 2.3.4. Experiments with 10 h Elimination of Bile Flow to the Duodenum

To assess the role of bile in the regulation of pancreatic exocrine secretion, experiments were performed on six piglets surgically prepared according to Experimental Model II.

In the control condition (Experiment 22; Appendix A.1, Table A3), both pancreatic juice and bile were returned to the duodenum overnight, and disconnection of the pancreatic and bile catheters occurred only during the experimental observation period.

In subsequent experiments (Experiments 23–26), bile flow to the duodenum was eliminated for 10 h prior to the experiment. In Experiment 23, the effect of prolonged bile diversion (10 h before the experiment and 5 h during the experimental period) on pancreatic juice secretion was evaluated.

In Experiments 24–26, pancreatic juice secretion was assessed under conditions of eliminated bile flow during intraduodenal infusion of exogenous bile acid salt solutions. Three concentrations of bile acid salts were tested: 0.02 M (Experiment 24), 0.04 M (Experiment 25), and 0.08 M (Experiment 26), administered at a constant rate of 7.5 mL/kg body mass/hour (Appendix A.1, Table A3).

#### 2.3.5. Experimental Scheme and Bile Acid Salt Infusions Under Interrupted Bile Flow

The experimental scheme for studies with preserved or interrupted bile flow to the duodenum (Experiments 22–26) is summarised in Appendix A.1 (Table A3), and the detailed protocol design is illustrated in Appendix A.2 (Scheme A3).

All experiments lasted 5 h and comprised a 1 h pre-feeding phase, a 1 h feeding phase, and a 3 h post-feeding observation period, conducted under conditions of either preserved or eliminated bile flow to the duodenum.

Pancreatic juice and bile were collected in the same manner as described for previous experiments. After sample collection for biochemical analyses, pancreatic juice was returned to the duodenum in all experimental conditions, whereas bile was returned only in control experiments.

Under conditions of eliminated bile flow, intraduodenal infusions of bile acid salt solutions were performed to assess concentration-dependent effects on pancreatic juice secretion. Infusions began with the onset of the feeding phase and continued until the end of the first hour of the post-feeding phase (Appendix A.2, Scheme A3).

Bile acid salt solutions were prepared using sodium salts of glycocholic acid, cholic acid, and deoxycholic acid derived from bovine bile (Sigma-Aldrich, St. Louis, MO, USA). Solutions with final concentrations of 0.02 M, 0.04 M, and 0.08 M were prepared by dissolving the appropriate amounts of bile acid salts in physiological saline and were administered intraduodenally at a constant rate of 7.5 mL/kg body mass/hour.

### 2.4. Analysis of Pancreatic Juice and Bile

#### 2.4.1. Determination of Total Protein Content and Trypsin Activity in Pancreatic Juice

In all the studies performed, the volume of secreted pancreatic juice was measured, and the total protein content in it was determined by the Lowry et al. method, modified and adapted for analysis in microplates [22]. Bovine serum albumin (BSA—Bovine serum albumin, A-7638, Sigma Chemicals, Co., St. Louis, MO, USA) was used as the standard.

Trypsin activity was determined after activation of pancreatic juice with enterokinase (Sigma Chemicals, Co., USA) and using the BAPNA substrate (Na-benzoyl-DL-arginine-p-nitroanilide, Sigma) according to the method of Erlanger et al. adapted for microplate analysis [22,28]. The release of p-nitroaniline was monitored by measuring absorbance at a wavelength of 405 nm. Enzyme activity was expressed in units (U), defined as the amount of enzyme catalysing the hydrolysis of 1 µmol of substrate per minute [29].

#### 2.4.2. Determination of Total Leptin Concentration in Bile

Leptin concentration in bile samples was determined by radioimmunoassay (RIA) using the Multi-Species Leptin RIA Kit (LINCO Research, Inc., St. Charles, MO, USA), which contains ^125^I-labelled human leptin, primary antibody (anti-Multi-Species Leptin) from guinea pig serum, secondary antibody (anti-Guinea Pig IgG) from goat serum, 3% PEG, recombinant human leptin standards, and buffers. The antibodies are recommended for determining leptin in plasma of many species, including pigs (the lowest leptin concentration detected by the test is 1.0 ng/mL, and the antibodies show 67% affinity for porcine leptin). The radioactivity of standards and samples was analysed in a Packard–Cannbera gamma counter. Based on the readings of the standards, the counter automatically calculated the leptin concentration in the analysed samples.

### 2.5. Determination of Cholecystokinin Concentration in Blood

Blood samples of approximately 5 mL were collected into tubes containing K_3_EDTA (tripotassium ethylenediaminetetraacetic acid, Sigma) and aprotinin (Traskolan, Jelfa, Jelenia Góra, Poland) 15 min before the feeding period and 15 min before the end of the feeding period. After centrifugation, plasma was collected and stored at –20 °C for CCK analysis. Cholecystokinin was determined in ethanol extracts using a ready-made radioimmunoassay kit (CCK RIA-Kit, Eurodiagnostica, Stockholm, Sweden) based on the double-antibody method. CCK-8s was used as the standard. The minimum sensitivity of the test was 0.3 pmol/L, and the intra-assay error was 6%. CCK was measured at pre-feeding and late-feeding time points.

### 2.6. Calculations and Statistical Analysis

Pancreatic juice secretion (volume, total protein concentration, and trypsin activity) and bile volume were expressed as output parameters and standardised to body mass (mL/kg body mass/h, mg/kg body mass/h, U/kg body mass/h, and mL/kg body mass/h, respectively). Pancreatic juice secretion during the perifeeding period, under conditions of either the presence or absence of pancreatic juice flow to the duodenum and following administration of pancreatin solutions with different trypsin activities (20%, 100%, and 200%), was expressed as the difference in secretion between the feeding and pre-feeding phases and between the post-feeding and pre-feeding phases. Data are presented as mean values *n* = 6 ± standard errors (SE).

After checking the normality of the data distribution using the Shapiro–Wilk test, statistical significance was analysed using the Friedman test (a nonparametric equivalent of one-way analysis of variance for repeated measures) and, if appropriate, the Wilcoxon test for dependent variables. Statistically significant differences were considered at a significance level of *p* < 0.05. Statistical analyses were performed using Statistica version 5.1 (StatSoft, Tulsa, OK, USA).

## 3. Results

**Figure 2:**
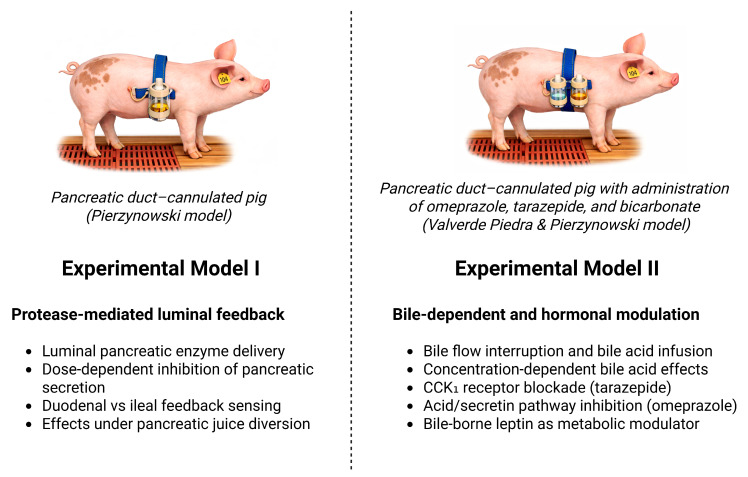
Overview of Experimental Models I and II and main experimental manipulations. Created using BioRender [30].

Detailed protocol variants are summarised in Appendix A. For clarity, the Results are organised by regulatory question rather than by protocol number. Section 3.1, Section 3.2 and Section 3.3 report findings from Experimental Models I and II (Figure 2), respectively, focusing on evidence directly relevant to the primary feedback mechanisms.

### 3.1. Effect of Pancreatic Enzymes in the Small Intestine on Pancreatic Juice Secretion

#### 3.1.1. Pancreatic Juice Secretion Under Control and Interrupted Flow Conditions

Under control conditions with preserved pancreatic juice flow to the duodenum, basal pre-prandial pancreatic secretion was characterised by low pancreatic juice volume, protein output, and trypsin activity. Interruption of pancreatic juice delivery to the duodenum resulted in a significant increase in protein output and trypsin activity, while pancreatic juice volume showed only a numerical elevation (Table 2).

During the feeding phase, elimination of pancreatic juice flow led to a marked stimulation of pancreatic secretion, reflected by significantly higher pancreatic juice volume and protein output compared with preserved flow conditions. In the postprandial phase, pancreatic juice diversion maintained significantly elevated pancreatic juice volume and trypsin activity. Under preserved flow conditions, feed intake significantly stimulated protein and trypsin output, whereas pancreatic juice volume was not significantly affected (Table 2).

Plasma cholecystokinin (CCK) concentrations tended to be higher during interrupted pancreatic juice flow; however, due to high inter-individual variability, these differences did not reach statistical significance.

#### 3.1.2. Effect of Intraduodenal Pancreatin Infusion Under Preserved Pancreatic Juice Flow

Under preserved pancreatic juice flow, feeding induced a physiological increase in pancreatic juice secretion. Intraduodenal administration of pancreatin solutions corresponding to 20%, 100%, and 200% of feeding-phase trypsin activity produced a dose-dependent tendency toward attenuation of the feeding-induced secretory response; however, these effects did not reach statistical significance during the feeding phase (Table 3).

In the post-feeding phase, pancreatin infusion at 100% and 200% trypsin activity resulted in significantly higher protein output and trypsin activity compared with control conditions, with values exceeding those observed during the feeding phase.

#### 3.1.3. Effect of Intraduodenal Pancreatin Infusion Under Interrupted Pancreatic Juice Flow

When pancreatic juice flow was interrupted, feeding produced a pronounced stimulation of pancreatic secretion. Intraduodenal pancreatin infusion significantly reduced this response in a concentration-dependent manner. Administration of pancreatin at 100% trypsin activity markedly suppressed protein output and trypsin activity compared with untreated animals (Table 4).

Lower-dose pancreatin (20%) induced partial inhibition, whereas the highest dose (200%) showed reduced inhibitory efficacy. During the post-feeding phase, no significant differences among treatment groups were observed.

#### 3.1.4. Effect of Ileal Pancreatin Infusion Under Preserved Pancreatic Juice Flow

Under preserved pancreatic juice flow, ileal infusion of pancreatin at all tested concentrations did not significantly alter pancreatic secretion during the feeding phase. In the post-feeding phase, a dose-dependent tendency toward increased protein output and trypsin activity was observed, reaching statistical significance only at the highest pancreatin concentration (200%) (Table 5).

Plasma CCK concentrations were not significantly affected by ileal pancreatin administration under these conditions.

#### 3.1.5. Effect of Ileal Pancreatin Infusion Under Interrupted Pancreatic Juice Flow

Under conditions of interrupted pancreatic juice flow, ileal pancreatin infusion at all tested concentrations significantly reduced feeding-induced pancreatic enzyme secretion compared with untreated animals (Table 6). Protein output and trypsin activity were suppressed to levels comparable to those observed under preserved pancreatic juice flow.

The lowest pancreatin dose (20%) was sufficient to normalise pancreatic secretion. During the post-feeding phase, a significant reduction in protein output was observed at the 20% dose, whereas higher concentrations showed only numerical trends.

#### 3.1.6. Cholecystokinin Concentration in Peripheral Blood Plasma

Across all experimental conditions, plasma CCK concentrations did not differ significantly between preserved and interrupted pancreatic juice flow. Although higher mean CCK values were observed during interrupted flow, particularly in the feeding phase, substantial inter-individual variability prevented statistical confirmation. Neither duodenal nor ileal pancreatin infusion produced significant alterations in circulating CCK levels (Table 5 and Table 6).

### 3.2. Effect of Bile, Bicarbonate, and Pharmacological Interventions on Pancreatic and Bile Secretion

#### 3.2.1. Pancreatic Juice and Bile Secretion with Bile Flow Interruption and Bicarbonate Infusion

Under control conditions with preserved pancreatic juice and bile flow, pre-feeding pancreatic juice volume was 1.62 ± 0.20 mL/kg body mass/hour, protein output was 4.64 ± 1.33 mg/kg body mass/hour, and trypsin activity reached 2.44 ± 0.68 U/kg body mass/hour.

Interruption of bile flow to the duodenum significantly increased pancreatic juice volume to 3.00 ± 0.18 mL/kg body mass/hour and trypsin activity to 4.16 ± 0.28 U/kg body mass/hour (*p* < 0.05). In contrast, pancreatic protein output remained comparable to control values. Combined interruption of both bile and pancreatic juice flow produced a similar stimulatory effect on pancreatic secretion (Table 7).

During the feeding phase, bile flow interruption resulted in significantly higher pancreatic protein output (18.8 ± 2.13 mg/kg versus 12.9 ± 1.96 mg/kg in controls, *p* < 0.05) and increased trypsin activity (11.2 ± 1.14 U/kg versus 7.67 ± 1.49 U/kg, *p* < 0.05). Intraduodenal bicarbonate infusion under conditions of bile diversion showed a tendency toward further stimulation of pancreatic secretion, although these changes did not consistently reach statistical significance.

In the post-feeding phase, interruption of bile flow alone or combined bile and pancreatic juice diversion maintained elevated pancreatic secretion. The highest protein output was observed under combined interruption conditions (17.1 ± 2.48 mg/kg versus 8.03 ± 2.50 mg/kg in controls, *p* < 0.05) (Table 7).

#### 3.2.2. Effect of Gastric Acid Inhibition and CCK1 Receptor Blockade

Intravenous administration of omeprazole (1 mg/kg body mass) 30 min before feeding did not significantly affect pre-feeding pancreatic secretion. However, a tendency toward reduced trypsin activity and increased pancreatic juice volume was observed.

Intraduodenal administration of tarazepide (5 mg/kg body mass), a selective CCK_1_ receptor antagonist, tended to reduce pancreatic protein output and trypsin activity, while bile secretion showed a tendency to increase. Combined administration of omeprazole and tarazepide resulted in higher pancreatic juice volume accompanied by lower protein output and trypsin activity during the pre-feeding phase (Table 7).

During the feeding phase, omeprazole reduced pancreatic juice volume to 2.51 ± 0.43 mL/kg compared with 3.82 ± 0.43 mL/kg in control animals. Tarazepide caused a more pronounced reduction in pancreatic juice volume (2.78 ± 0.55 mL/kg) and was associated with a marked decrease in protein output (5.89 ± 0.93 mg/kg versus 12.9 ± 1.96 mg/kg in controls). Combined treatment further reduced pancreatic juice volume as well as protein and trypsin output. A significant reduction in bile flow was observed only following combined omeprazole and tarazepide administration (2.99 ± 0.36 mL/kg versus 3.96 ± 0.54 mL/kg in controls, *p* < 0.05).

In the post-feeding phase, all pharmacological treatments resulted in lower pancreatic secretion compared with control animals. The lowest pancreatic juice volume was observed following combined omeprazole and tarazepide treatment (0.92 ± 0.17 mL/kg). Under these conditions, bile flow was also significantly reduced (2.23 ± 0.20 mL/kg versus 4.05 ± 0.70 mL/kg in controls, *p* < 0.05) (Table 7).

### 3.3. Effect of Bile Flow Interruption and Bile Acid Salt Infusion on Pancreatic and Bile Secretion

#### 3.3.1. Effect of Prolonged Bile Flow Interruption

In experiments with extended observation, control pancreatic juice secretion during the pre-feeding phase averaged 1.54 ± 0.20 mL/kg body mass/hour, with protein output of 3.41 ± 0.36 mg/kg body mass/hour.

Interruption of bile flow for 10 h significantly increased pancreatic juice volume to 3.29 ± 0.37 mL/kg body mass/hour and protein output to 5.85 ± 0.85 mg/kg body mass/hour (*p* < 0.05). At the same time, bile secretion was significantly reduced under interrupted conditions (1.94 ± 0.27 mL/kg body mass/hour versus 2.36 ± 0.26 mL/kg in controls) (Table 8).

During the feeding phase, pancreatic juice volume increased from 3.83 ± 0.45 mL/kg body mass/hour under control conditions to 4.89 ± 0.34 mL/kg body mass/hour during bile diversion. Although this increase did not reach statistical significance, protein output and trypsin activity were markedly elevated (32.0 ± 7.12 mg/kg versus 14.6 ± 1.67 mg/kg and 24.5 ± 7.79 U/kg versus 6.97 ± 0.97 U/kg, respectively; both *p* < 0.05). In parallel, bile secretion was strongly suppressed to 1.17 ± 0.17 mL/kg body mass/hour (*p* < 0.05).

In the first hour following feeding, pancreatic juice volume remained numerically higher under bile flow interruption (4.29 ± 0.45 mL/kg versus 2.43 ± 0.36 mL/kg), while protein output and trypsin activity were significantly increased compared with control animals (24.7 ± 5.07 mg/kg versus 11.3 ± 1.74 mg/kg and 16.8 ± 4.86 U/kg versus 5.33 ± 0.62 U/kg, respectively; *p* < 0.05). Bile secretion remained significantly reduced (1.76 ± 0.23 mL/kg versus 3.67 ± 0.40 mL/kg, *p* < 0.05).

During the second and third post-feeding hours, pancreatic juice volume remained elevated under bile diversion (3.87 ± 0.38 and 3.34 ± 0.42 mL/kg body mass/hour, respectively). Protein output remained significantly higher throughout this period (20.6 ± 4.25 mg/kg in hour two and 22.9 ± 3.81 mg/kg in hour three, *p* < 0.05). Trypsin activity showed sustained elevation, although statistical significance was not reached at time points. Bile secretion remained significantly suppressed across all post-feeding hours (Table 8).

#### 3.3.2. Effect of Intraduodenal Bile Acid Salt Infusion

Intraduodenal infusion of bile acid salt solutions at concentrations of 0.02 M, 0.04 M, and 0.08 M under conditions of interrupted bile flow modulated pancreatic secretion in a concentration-dependent manner.

During the feeding phase, infusion of 0.02 M and 0.04 M bile acid solutions resulted in significantly increased pancreatic protein output and trypsin activity compared with pre-feeding values (*p* < 0.05). In contrast, administration of the highest concentration (0.08 M) failed to stimulate pancreatic secretion in response to food intake (Table 8).

In the post-feeding phase, pancreatic protein output and trypsin activity displayed clear dose-dependent patterns. Animals receiving the 0.02 M solution maintained significantly elevated secretion throughout the 3 h observation period (*p* < 0.05). In contrast, infusion of 0.04 M bile acids resulted in partial attenuation of postprandial secretion, with values approaching pre-feeding levels. The highest bile acid concentration (0.08 M) produced suppression of the postprandial pancreatic secretory response.

Bile volume did not differ significantly among treatment groups during the feeding phase. However, during the post-feeding period, bile outflow increased significantly, with the highest concentrations producing the greatest elevations. The 0.08 M solution induced the most pronounced postprandial increase in bile secretion, reaching statistical significance during post-feeding hours one to three (Table 8).

### 3.4. Leptin Concentration in Bile

Leptin concentration in bile under control conditions, with preserved pancreatic juice and bile flow to the duodenum, ranged between 16.5 and 19.0 ng/mL and did not differ significantly between interdigestive and digestive periods. Interruption of bile flow resulted in lower biliary leptin concentrations, ranging from 11.7 to 14.5 ng/mL.

During the interdigestive period, leptin secretion in control animals averaged 40.6 ± 12.2 ng/kg body mass/hour. During the feeding phase, leptin secretion increased to 75.4 ± 24.7 ng/kg body mass/hour, although this increase did not reach statistical significance. Elevated leptin secretion was maintained during the first and second post-feeding hours (69.5 ± 17.5 and 62.4 ± 5.5 ng/kg body mass/hour, respectively).

Under conditions of interrupted bile flow, leptin secretion during the feeding phase was significantly reduced to 15.3 ± 5.7 ng/kg body mass/hour (*p* < 0.05), corresponding to an approximately 50% decrease compared with control values. In the first and second hours following feeding, leptin secretion remained significantly lower under bile diversion (23.0 ± 10.4 and 20.0 ± 7.4 ng/kg body mass/hour, respectively; *p* < 0.05). A gradual recovery toward baseline values was observed during the third post-feeding hour (Table 9).

## 4. Discussion

The present study evaluates several feedback mechanisms involved in the regulation of pancreatic juice secretion in piglets and provides integrated physiological observations on the interactions between pancreatic enzyme-dependent feedback, bile acid-related signalling, hormonal regulation, and metabolic influences [1,2,31]. These observations extend existing concepts of exocrine pancreatic regulation, which have been derived largely from rodent models [1,32,33], by examining these mechanisms in a large-animal model that displays closer physiological similarity to humans, particularly with regard to migrating motor complex (MMC) periodicity and patterns of pancreatic enzyme secretion [34,35,36].

Taken together, the results support the interpretation that pancreatic exocrine secretion is governed by multiple interacting regulatory pathways. These include protease-dependent luminal feedback involving trypsin-mediated inactivation of CCK-releasing peptides, as well as modulatory influences related to bile acids, hormone-dependent signalling (CCK and secretin), and metabolic factors such as leptin. Rather than acting as isolated mechanisms, these pathways appear to function in a coordinated and partially compensatory manner, allowing pancreatic secretion to adapt to alterations in individual regulatory inputs. Given the breadth of experimental variants, the discussion is intentionally focused on findings that directly inform the central feedback mechanisms governing pancreatic exocrine regulation.

### 4.1. The Importance of Pancreatic Juice in Feedback Regulation of the Exocrine Pancreas

Interruption of pancreatic juice flow to the duodenum was associated with a marked increase in pancreatic enzyme secretion, reflected by elevated protein output and trypsin activity, particularly during the preprandial phase. This response is consistent with the classical concept of protease-dependent negative feedback, whereby luminal trypsin limits pancreatic secretion through proteolytic inactivation of cholecystokinin (CCK)-releasing factor (LCRF) and monitor peptide (MP) [4,6,37,38]. Under physiological conditions, the return of pancreatic juice to the duodenum promotes degradation of these peptides, thereby reducing stimulation of intestinal I cells and limiting further enzyme release [1,37,38,39]. Disruption of this mechanism permits accumulation of CCK-releasing peptides and consequently enhances pancreatic secretion.

Despite the pronounced stimulation of enzyme output following pancreatic juice diversion, circulating plasma CCK concentrations exhibited high inter-individual variability and did not reach statistical significance under several experimental conditions. This dissociation between peripheral CCK levels and pancreatic secretory responses highlights a limitation inherent to endocrine measurements in large-animal models. Given the short plasma half-life of CCK and the predominant role of paracrine and vagally mediated signalling, circulating CCK concentrations may not accurately reflect functional activation of CCK-dependent regulatory pathways at the intestinal–neural interface [5,6,40].

Further support for a protease-dependent feedback mechanism was provided by the dose-dependent inhibition observed during intraduodenal pancreatin administration. Maximal suppression of pancreatic protein output occurred at pancreatin doses corresponding to physiological trypsin activity, whereas supraphysiological concentrations were associated with partial recovery of secretion. This biphasic response suggests saturable feedback kinetics and may reflect depletion of luminal CCK-releasing peptides, receptor desensitisation, or recruitment of compensatory regulatory pathways. Such behaviour is consistent with current concepts that protease-dependent feedback constitutes one component of a broader, non-linear regulatory network rather than a simple on–off mechanism [1,2,31].

A notable finding of the present study is the effectiveness of protease-dependent feedback at distal intestinal sites. Ileal delivery of pancreatin under conditions of interrupted pancreatic juice flow significantly reduced pancreatic enzyme secretion to levels comparable with those observed during preserved duodenal return. This observation challenges the traditional duodenum-centred view of pancreatic feedback regulation and supports the concept of distributed enteroendocrine sensing along the length of the small intestine [5,34,39]. The sensitivity of ileal feedback, particularly at relatively low pancreatin doses, suggests that protease-dependent regulation is not confined to proximal intestinal segments.

These findings are compatible with an extension of the ileal brake concept beyond its established role in gastrointestinal motility and nutrient absorption to include modulation of pancreatic exocrine secretion. From a translational perspective, this mechanism may be relevant in clinical settings such as pancreatic enzyme replacement therapy, where distal delivery of active enzymes could contribute to suppression of endogenous pancreatic secretion [5,39].

Pharmacological inhibition of CCK_1_ receptors provided additional evidence for the involvement of CCK-dependent pathways in pancreatic enzyme regulation. Administration of tarazepide resulted in a substantial reduction in prandial enzyme output, indicating that a significant proportion of food-stimulated pancreatic secretion in piglets depends on CCK_1_-mediated signalling. Given the reported predominance of CCK_2_ receptors on porcine acinar cells, this effect is most likely mediated indirectly via CCK_1_ receptors located on vagal afferent fibres, consistent with the established role of neural amplification mechanisms in large mammals [1,19,41,42,43].

In contrast, inhibition of gastric acid secretion with omeprazole primarily reduced pancreatic juice volume without significantly affecting enzyme output. This pattern supports the established role of acid-stimulated secretin release in regulating ductal bicarbonate secretion while indicating that pancreatic enzyme output is governed predominantly by CCK-dependent and neural mechanisms rather than by acid–secretin signalling alone [39,44].

The most pronounced inhibitory effect on pancreatic enzyme secretion was observed following combined blockade of gastric acid secretion and CCK_1_ receptors. The additive nature of this suppression indicates that pancreatic exocrine secretion is controlled by parallel regulatory pathways rather than by a single dominant mechanism [13,16,38]. The concurrent reduction in bile output further suggests coordinated regulation of pancreatic enzyme secretion and gallbladder emptying, ensuring synchronised delivery of digestive enzymes and bile salts during nutrient digestion.

### 4.2. The Importance of Bile in Feedback Regulation of the Exocrine Pancreas

One of the most prominent observations of the present study was the sustained stimulation of pancreatic enzyme secretion following interruption of bile flow to the duodenum. Under these conditions, both pancreatic protein output and trypsin activity increased markedly during the prandial phase and remained elevated throughout the postprandial period. This prolonged hypersecretion indicates that bile-derived signals exert an inhibitory influence on pancreatic exocrine secretion under physiological conditions.

Previous experimental studies have suggested an inhibitory role of bile acids in the regulation of pancreatic enzyme secretion; however, the magnitude and physiological relevance of this effect have varied considerably between species and experimental models. The present findings are consistent with the concept that physiological exposure to bile acids provides a tonic suppressive signal limiting excessive pancreatic enzyme secretion, whereas removal of this signal leads to compensatory hypersecretion. In this context, bile acids may contribute to feedback regulation by adjusting pancreatic output once lipid digestion has been initiated [13,42,45].

Infusion of bile acid salts at graded concentrations revealed a clear concentration-dependent modulation of pancreatic secretion. Lower concentrations (0.02 M) maintained or modestly enhanced enzyme output, whereas higher concentrations (0.08 M) markedly suppressed postprandial pancreatic secretion. Intermediate concentrations (0.04 M) produced transitional responses between these two extremes. This biphasic pattern indicates that bile acids do not function as simple inhibitory signals but rather modulate pancreatic secretion in a dose-dependent manner [44,46,47].

Several bile acid–responsive signalling pathways have been proposed to mediate such effects, including the farnesoid X receptor (FXR/NR1H4) and the Takeda G-protein–coupled bile acid receptor (TGR5/GPBAR1). Activation of FXR in intestinal epithelial cells has been linked to induction of fibroblast growth factor 15/19 (FGF15 in rodents, FGF19 in humans), whereas TGR5 activation has been associated with modulation of enteroendocrine signalling. Importantly, neither FXR nor TGR5 signalling was directly assessed in the present study, and their involvement should therefore be regarded as hypothetical. The present results demonstrate a physiological phenomenon rather than a defined molecular mechanism [13,35,45,48].

The bile acid concentrations applied in this study (20–80 mM) approximate luminal levels encountered in the proximal small intestine following postprandial gallbladder emptying and exceed systemic portal concentrations by one to two orders of magnitude. This spatial gradient is physiologically relevant, as intestinal epithelial cells, enteroendocrine cells, and pancreatic ductal epithelium are capable of sensing luminal bile acid exposure independently of systemic bile acid signalling [11,46].

Collectively, these findings demonstrate that bile acids modulate pancreatic exocrine secretion in a concentration-dependent manner in vivo. While the underlying receptor mechanisms remain unresolved, the results provide a functional framework supporting bile acids as modulatory signals within the multi-level feedback system governing pancreatic secretion [14,35,45,48]. Future studies employing receptor-selective pharmacological tools and direct measurement of downstream signalling pathways will be required to delineate the relative contributions of FXR-, TGR5-, and alternative mechanisms.

### 4.3. Leptin in Bile

The present study provides novel observational data on leptin concentrations in bile under different experimental conditions. To our knowledge, biliary leptin secretion has not previously been characterised in a chronic large-animal model. The results demonstrate a temporal association between changes in biliary leptin output and alterations in pancreatic exocrine secretion; however, no causal relationship can be inferred from the present data [19,49,50].

Leptin is primarily recognised as a metabolic hormone involved in central regulation of energy balance and glucose homeostasis. Experimental studies have demonstrated that leptin-responsive neuronal pathways, including projections to the dorsal motor nucleus of the vagus, may link metabolic status with pancreatic endocrine and exocrine function. This provides a conceptual framework for indirect modulation of pancreatic activity by metabolic signals, although such mechanisms were not directly addressed in the present study. In vitro and ex vivo studies have suggested that leptin may exert concentration-dependent effects on pancreatic enzyme secretion. Physiological leptin concentrations have been reported to potentiate CCK-stimulated acinar responses, whereas supraphysiological levels may inhibit enzyme secretion through CCK1 receptor-dependent vagal mechanisms. Importantly, these observations originate primarily from isolated tissue and cell culture models and cannot be directly extrapolated to the in vivo conditions applied here [6,9,18,19,42,43].

In the current study, interruption of bile flow resulted in an approximately 50% reduction in biliary leptin output, concomitant with a marked increase in pancreatic enzyme secretion. Although this inverse relationship is intriguing, it remains purely correlative. Several alternative mechanisms may account for pancreatic hypersecretion during bile diversion, including loss of bile acid–dependent inhibitory signalling, altered intestinal luminal conditions, changes in CCK release kinetics, and simultaneous disruption of multiple feedback pathways [13,36,42,45].

Accordingly, the present findings should be regarded as hypothesis-generating rather than mechanistically conclusive. Biliary leptin may represent a metabolic signal associated with bile-dependent regulation of digestive processes rather than a primary regulator of pancreatic exocrine secretion. Future studies employing direct functional manipulation of leptin signalling, combined with assessment of bile acid receptor pathways, will be required to determine whether leptin contributes to phase-dependent fine-tuning of pancreatic exocrine function.

The experimental findings are consistent with a hierarchically organised framework of pancreatic feedback regulation, in which protease-dependent luminal feedback represents the dominant inhibitory component, while bile- and metabolism-related pathways exert modulatory influences on pancreatic exocrine secretion (Table 10).

### 4.4. Species Specific Considerations: Pigs Versus Rodents and Humans

The pig represents an intermediate translational model between rodents and humans with respect to pancreatic exocrine physiology. This is particularly evident in the periodicity of the migrating motor complex (MMC) and its temporal coordination with pancreatic secretion. In contrast to rodents, which exhibit short MMC cycles (~20 min), and humans, in whom cycles typically last ~90 min, pigs display an intermediate periodicity of approximately 60 min. This temporal organisation allows investigation of regulatory mechanisms operating over longer physiological time scales, which may not be adequately captured in rodent models [44,51,52]. Importantly, several key features of pancreatic exocrine regulation in pigs, including meal size, bile acid composition, and the organisation of postprandial secretory phases, more closely resemble human physiology than rodent models, supporting the translational relevance of porcine findings.

The robust negative feedback responses observed in the present study support the physiological relevance of protease-dependent regulation in large mammals. Moreover, the finding that both duodenal and ileal administration of pancreatin effectively suppressed pancreatic enzyme secretion suggests that feedback sensing is not restricted to the proximal intestine but may operate along an extended segment of the small intestine. This observation is consistent with the concept of distributed enteroendocrine sensing of luminal proteolytic activity and may contribute to species-specific differences reported in the literature [16,34,44,52]. Such distributed sensing mechanisms may be particularly relevant to human conditions characterised by altered intraluminal protease activity, including pancreatic exocrine insufficiency, enzyme replacement therapy, and post-surgical alterations of intestinal transit.

Despite these translational advantages, caution is required when extrapolating pharmacological findings to humans. Species-specific differences in drug absorption, hepatic metabolism, receptor expression, and neural organisation may substantially influence the magnitude and mechanisms of pancreatic responses. In particular, pigs have been reported to exhibit predominant CCK-2 receptor expression on pancreatic acinar cells, whereas humans display higher CCK-1 receptor expression on both acinar cells and vagal afferent terminals [9,15,23,53,54].

In the present study, pharmacological blockade with the CCK1 receptor antagonist tarazepide resulted in approximately 54% suppression of food-stimulated pancreatic enzyme secretion. This finding suggests a significant contribution of CCK1-dependent signalling in piglets, likely mediated predominantly through indirect vagal pathways. However, the relative contribution of direct acinar versus neural mechanisms cannot be resolved from the current data [9,25]. Nevertheless, the demonstration of substantial CCK1-dependent modulation of pancreatic secretion in a large-animal model underscores the potential relevance of CCK-mediated neural pathways in human postprandial pancreatic regulation.

Accordingly, quantitative effects observed in pigs should not be directly extrapolated to humans. Prospective human studies employing analogous experimental designs, together with receptor-selective antagonists and targeted neural interventions, will be required to delineate species-specific regulatory pathways and to refine the translational relevance of these findings.

### 4.5. Study Limitations

Several limitations of the present study should be acknowledged.

First, although CCK-dependent feedback constituted an important working hypothesis, circulating plasma CCK concentrations did not show statistically significant changes under several experimental conditions. This likely reflects the short plasma half-life of CCK, its pulsatile secretion pattern, and the considerable inter-individual variability characteristic of large-animal models. Moreover, peripheral plasma CCK levels may not accurately reflect local paracrine signalling within the intestinal mucosa, where CCK primarily activates vagal afferent pathways involved in pancreatic exocrine regulation. Accordingly, the absence of significant endocrine CCK responses does not exclude functional involvement of CCK-dependent neural mechanisms.

Second, the observed regulatory effects on pancreatic secretion may involve CCK-independent pathways, including enteric and vagal reflex circuits as well as bile acid–dependent signalling. Although bile acid receptors such as FXR and TGR5 were not directly assessed in the present study, their established roles in enteroendocrine and metabolic regulation suggest that they may contribute to the responses observed following bile diversion and bile acid supplementation. These pathways warrant direct investigation in future mechanistic studies.

Third, trypsin activity in pancreatic juice was assessed using a colorimetric assay, which allows reliable relative comparisons between experimental conditions but offers limited analytical sensitivity at low enzyme activities. Fluorescent substrate–based assays provide improved sensitivity and dynamic range (PMID: 31899136; PMID: 38553043) and may represent a valuable methodological alternative for future studies focusing on protease-dependent feedback mechanisms.

Finally, the relatively small number of animals per experimental condition (*n* = 6 or fewer) reflects the technical complexity, ethical constraints, and long-term nature of the chronic cannulation model employed. Importantly, each animal served as its own control within a repeated-measures design, substantially reducing inter-individual variability and increasing statistical efficiency despite limited group sizes. Given the small sample size and non-normal distribution of several outcome variables, nonparametric tests for repeated measures (the Friedman test followed by the Wilcoxon signed-rank test) were considered the most appropriate statistical approach and are widely accepted in physiological studies using large-animal models.

No formal correction for multiple comparisons was applied, as the analyses were hypothesis-driven and focused on predefined, physiologically justified contrasts rather than exploratory screening of multiple endpoints. Application of conservative correction procedures under these conditions would markedly increase the risk of type II error and obscure biologically relevant regulatory effects.

The relatively large standard errors observed for some variables reflect inherent biological variability associated with in vivo digestive secretory responses, particularly in large-animal models. Importantly, consistent directional changes across experimental conditions and convergence of multiple independent outcome measures (pancreatic juice volume, protein output, trypsin activity, and bile secretion) support the physiological relevance of the observed effects despite variability in absolute values.

## 5. Conclusions

This study provides experimental evidence that pancreatic exocrine secretion in weaned piglets is regulated by multiple interacting feedback mechanisms operating along the gastrointestinal tract. Using chronic porcine models, we demonstrate that pancreatic enzyme output is shaped by the integrated action of luminal, hormonal, and bile-related signals.

Protease-dependent luminal feedback emerged as a dominant inhibitory component, as interruption of pancreatic juice flow consistently stimulated enzyme secretion across physiologically relevant protease concentrations and at both proximal and distal intestinal sites. Bile and bile acids acted as modulatory signals, with bile diversion producing sustained stimulation of pancreatic secretion, indicating a tonic inhibitory influence on exocrine output. The specific receptor pathways underlying bile acid–mediated effects remain to be elucidated.

Hormonal pathways involving cholecystokinin and secretin contributed through partially parallel mechanisms, as combined pharmacological blockade resulted in additive suppression of pancreatic secretion. In addition, bile-borne metabolic signals, including leptin, were associated with phase-dependent changes in pancreatic secretion, suggesting a potential modulatory role, although causal relationships were not established.

Overall, the findings support an integrative regulatory framework in which protease-dependent feedback constitutes a core control mechanism, while bile-, hormone-, and metabolism-related signals modulate the magnitude and temporal pattern of pancreatic exocrine secretion.

## Figures and Tables

**Table 1:** Composition of the commercial feed mixture for piglets used in the experiments.

Composition	Content
Metabolizable Energy (Mj·Kg^−1^)	12.2
Total Protein (%)	15.5
Fat (%)	4.5
Fibre (%)	5.0
Lysine (G·Kg^−1^)	9.9
Methionine (G·Kg^−1^)	3.6
Threonine (G·Kg^−1^)	5.9
Calcium (%)	0.75
Phosphorus (%)	0.55
Vitamin A (Iu)	8000
Vitamin D3 (Iu)	800

**Table 2:** Pancreatic juice (PJ) secretion and Cholecystokinin (CCK) concentration in blood plasma in piglets with preserved (PJ+) and eliminated (PJ−) pancreatic juice flow to the duodenum within pancreatic secretory phases (Values represent means *n* = 6 ± SE).

	Pancreatic Secretory Phase					
	Preprandial		Prandial		Postprandial	
	PJ+	PJ−	PJ+	PJ−	PJ+	PJ−
PJ output (mL/h/kg b.wt.)	1.71 ± 0.55	2.60 ± 0.16	3.20 ± 0.58 ^a^	4.59 ± 0.50 ^b^	2.76 ± 0.79 ^a^	4.99 ± 0.41 ^b^
PJ protein output (mg/h/kg b.wt.)	2.78 ± 0.49 ^a^	4.87 ± 1.08 ^b^	9.07 ± 1.82 ^a^	28.2 ± 3.56 ^b^	4.43 ± 1.17	12.7 ± 3.72
PJ trypsin output (U/h/kg b.wt.)	2.04 ± 0.41 ^a^	4.87 ± 1.08 ^b^	5.63 ± 0.73	15.4 ± 2.62	2.89 ± 0.61 ^a^	7.69 ± 1.05 ^b^
CCK (pmol/L)	1.36 ± 0.54	2.85 ± 2.11	1.79 ± 0.02	5.34 ± 3.94	-	-

^a,b^—different letters indicate statistically significant differences between preserved and interrupted PJ duodenal flow in the respective pancreatic secretory phases at the level of *p* < 0.05. (Wilcoxon test).

**Table 3:** Pancreatic juice (PJ) secretion during the feeding and post-feeding phase in piglets under conditions of preserved pancreatic juice flow and administration of pancreatin solutions to the duodenum (20%, 100%, 200% of trypsin activity). Changes in pancreatic juice secretion is presented as increments between feeding phase, post-feeding phase and pre-feeding phase values, respectively (means *n* = 6 ± SE).

	Pancreatin Intraduodenal Infusions Under Preserved PJ Flow Conditions			
Pancreatic secretory phase	100% id. PJ + id pancreatin infusions			
	0%	20%	100%	200%
Pancreatic juice output (mL/h/kg b.wt.)				
Preprandial means	1.72 ± 0.12	0.98 ± 0.40	1.52 ± 0.27	1.44 ± 0.25
Prandial increments	1.48 ± 0.46	0.74 ± 0.52	0.20 ± 0.39	0.28 ± 0.39
Postprandial increments	1.05 ± 0.57	1.20 ± 0.63	1.02 ± 0.38	1.34 ± 0.72
Pancreatic juice protein output increments (mg/h/kg b.wt.)				
Preprandial means	2.70 ± 0.40	6.90 ± 1.49	5.63 ± 0.96	4.50 ± 0.05
Prandial increments	6.29 ± 2.18	4.09 ± 1.89	2.85 ± 1.36	1.74 ± 0.32
Postprandial increments	1.65 ± 1.58 ^a^	3.25 ± 0.92 ^a^	9.44 ± 3.01 ^b^	9.56 ± 4.00 ^b^
Pancreatic juice trypsin output increments (U/h/kg b.wt.)				
Preprandial means	2.04 ± 0.41	4.08 ± 0.54	3.86 ± 0.42	3.43 ± 0.12
Prandial increments	3.59 ± 0.98	2.04 ± 1.03	1.82 ± 0.83	1.39 ± 0.30
Postprandial increments	0.85 ± 0.81 ^a^	1.97 ± 0.49 ^a^	6.68 ± 2.61 ^b^	7.53 ± 3.41 ^b^

^a,b^ = *p* < 0.05—different letters indicate statistically significant differences between respective pancreatic secretory phases of preserved and interrupted PJ duodenal flow (Wilcoxon test.).

**Table 4:** Pancreatic juice (PJ) secretion during the feeding and post-feeding phase in piglets under conditions of interrupted pancreatic juice flow and administration of pancreatin solutions to the duodenum (20%, 100%, 200% of trypsin activity). Changes in pancreatic juice secretion are presented as increments between feeding phase, post-feeding phase and pre-feeding phase values, (means *n* = 6 ± SE).

	Pancreatin Intraduodenal Infusions Under Interrupted PJ Flow			
Pancreatic secretory phase	0% id. PJ + id pancreatin infusions			
	0%	20%	100%	200%
Pancreatic juice output (mL/h/kg b.wt.)				
Preprandial mean	2.60 ± 0.08	1.90 ± 0.45	2.34 ± 0.59	1.43 ± 0.82
Prandial increments	1.99 ± 0.58	0.70 ± 0.41	0.26 ± 0.56	1.17 ± 0.74
Postprandial increments	1.93 ± 1.06	1.00 ± 0.54	1.01 ± 0.67	0.47 ± 0.75
Pancreatic juice protein output increments (mg/h/kg b.wt.)				
Preprandial mean	11.6 ± 0.44	7.18 ± 2.42	10.1 ± 2.31	3.72 ± 1.31
Prandial increments	16.6 ± 4.00 ^A^	4.38 ± 2.16 ^A^^B^	1.46 ± 2.01 ^B^	7.84 ± 4.74 ^A^^B^
Postprandial increments	6.98 ± 2.02	3.06 ± 1.04	3.75 ± 1.45	3.20 ± 2.88
Pancreatic juice trypsin output increments (U/h/kg b.wt.)				
Preprandial mean	4.80 ± 0.54	0.88 ± 0.36	3.74 ± 1.93	0.05 ± 0.3
Prandial increments	10.6 ± 2.99 ^a^	3.92 ± 2.00 ^a^^b^	1.07 ± 1.50 ^b^	4.86 ± 3.25 ^a^^b^
Postprandial increments	4.43 ± 1.72	2.62 ± 0.86	3.13 ± 1.01	2.48 ± 2.56

^a,b^ = *p* < 0.05, ^A,B^ = *p* < 0.01. Different letters indicate statistically significant differences between treatments (Wilcoxon test).

**Table 5:** Pancreatic juice (PJ) secretion and cholecystokinin (CCK) concentration in peripheral blood plasma during the feeding and post-feeding phase in piglets under conditions of preserved pancreatic juice flow and administration of pancreatin solutions to the ileum (20%, 100%, 200% of trypsin activity). Changes in pancreatic juice secretion are presented as increments between feeding phase, post-feeding phase and pre-feeding phase values (means *n* = 6 ± SE).

	Pancreatin Intraileal Infusions Under Preserved PJ Flow Conditions			
Pancreatic secretory phase	100% id. PJ + Ileal pancreatin infusion			
	0%	20%	100%	200%
Pancreatic juice output (mL/h/kg b.wt.)				
Preprandial means	1.72 ± 0.12	1.40 ± 0.25	1.45 ± 0.34	1.72 ± 0.09
Prandial increments	1.48 ± 0.46	0.32 ± 0.38	0.27 ± 0.43	1.21 ± 0.27
Postprandial increments	1.05 ± 0.57	0.20 ± 0.62	0.90 ± 0.34	1.05 ± 0.98
Pancreatic juice protein output increments (mg/h/kg b.wt.)				
Preprandial means	2.78 ± 0.40	10.8 ± 3.24	10.5 ± 3.41	6.88 ± 0.42
Prandial increments	6.29 ± 2.18	8.06 ± 3.64	7.77 ± 3.81	4.11 ± 1.00
Postprandial increments	1.65 ± 1.58 ^a^	3.81 ± 1.45	5.23 ± 1.73	5.41 ± 2.48
Pancreatic juice trypsin output increments (U/h/kg b.wt.)				
Preprandial means	2.08 ± 0.41	7.04 ± 1.93	6.29 ± 2.28	4.56 ± 0.37
Prandial increments	3.59 ± 0.98	5.00 ± 2.33	4.25 ± 2.49	4.38 ± 2.26
Postprandial increments	0.85 ± 0.81 ^a^	2.73 ± 1.10 ^a^^b^	3.17 ± 1.25 ^a^^b^	4.38 ± 2.26 ^b^
Cholecystokinin (CCK) concentration in peripheral blood plasma (pmol/L)				
Prandial	1.36 ± 0.34	1.73 ± 0.31	2.62 ± 1.88	1.20 ± 0.27
Postprandial	1.79 ± 0.22	2.17 ± 0.81	2.60 ± 0.88	2.33 ± 0.80

^a,b^ = *p* < 0.05. Different letters indicate statistically significant differences between treatments (Wilcoxon test).

**Table 6:** Pancreatic juice (PJ) secretion during the feeding and post-feeding phase in piglets and cholecystokinin (CCK) concentration in peripheral blood plasma under conditions of interrupted pancreatic juice flow and administration of pancreatin solutions to the ileum (20%, 100%, 200% of trypsin activity). Pancreatic juice secretion is presented as increments between feeding phase, post-feeding phase and pre-feeding phase values (means *n* = 6 ± SE).

	Pancreatin Intraileal Infusions Under Interrupted PJ Flow			
	0% id PJ + ileal pancreatin infusion			
Pancreatic secretory phase	0%	20%	100%	200%
Pancreatic juice output (mL/h/kg b.wt.)				
Preprandial means	2.60 ± 0.08	1.73 ± 0.33	1.51 ± 0.63	2.57 ± 0.85
Prandial increments	1.99 ± 0.58	0.87 ± 0.27	1.08 ± 0.55	0.03 ± 0.78
Postprandial increments	1.93 ± 1.06	1.24 ± 0.38	1.13 ± 0.53	0.89 ± 0.84
Pancreatic juice protein output increments (mg/h/kg b.wt.)				
Preprandial means	11.6 ± 0.44	7.93 ± 2.09	6.84 ± 1.31	6.64 ± 1.75
Prandial increments	16.6 ± 4.00 ^A^	3.63 ± 1.30 ^b^	4.73 ± 2.87 ^b^	4.92 ± 3.07 ^b^
Postprandial increments	6.98 ± 2.02	2.02 ± 0.57 ^b^	6.38 ± 4.12 ^a^^b^	3.52 ± 3.40 ^a^^b^
Pancreatic juice trypsin output increments (U/h/kg b.wt.)				
Preprandial means	4.80 ± 0.54	3.23 ± 1.17	3.18 ± 0.80	2.81 ± 0.45
Prandial increments	10.6 ± 2.99 ^a^	1.58 ± 0.62 ^b^	1.63 ± 0.98 ^b^	1.99 ± 1.74 ^b^
Postprandial increments	4.43 ± 1.72	1.16 ± 0.32	3.38 ± 2.13	2.92 ± 2.08
Cholecystokinin (CCK) concentration in peripheral blood plasma (pmol/L)				
Prandial	2.85 ± 2.11	1.61 ± 0.47	2.90 ± 1.59	4.33 ± 0.57
Postprandial	5.34 ± 3.94	2.51 ± 0.78	3.39 ± 1.21	3.21 ± 0.78

^a,b^ = *p* < 0.05, ^A^ = *p* < 0.01. Different letters indicate statistically significant differences between treatments (Wilcoxon test).

**Table 7:** The Influence of interruption of bile and pancreatic juice duodenal flow and the effect of omeprazole iv. (OMP) and tarazepide id. (TA) administration on pancreatic juice secretion and bile outflow (*n* = 6 ± SEM).

Pancreatic Secretory Phase	Treatment					
	Bile+, PJ+ (Control)	Bile−, Pj+	Bile−, Pj−	Bile+, PJ+, OMP+	Bile+, PJ+, TA+	Bile+, PJ+, OMP+, TA+
Pancreatic juice output (mL/h/kg b.wt.)						
Preprandial	1.6 ± 0.3	2.8 ± 0.1 *	3.3 ± 0.8 *	2.5 ± 0.7	2.2 ± 0.5	1.3 ± 0.5
Prandial	3.3 ± 0.6	4.9 ± 0.9	4.8 ± 0.7	2.2 ± 0.6	2.9 ± 0.7	2.0 ± 0.8
Postprandial	2.4 ± 0.6	4.2 ± 0.7 *	4.1 ± 0.8 *	0.9 ± 0.1 *	2.0 ± 0.6	0.8 ± 0.1 *
Pancreatic juice protein output (mg/h/kg b.wt.)						
Preprandial	2.1 ± 0.5	6.7 ± 2.5	8.3 ± 3.0 *	4.2 ± 1.4	2.6 ± 1.4	2.6 ± 0.9
Prandial	9.7 ± 1.9	15.7 ± 5.6	16.0 ± 4.6	9.2 ± 3.7	5.4 ± 3.7 *	7.5 ± 1.9
Postprandial	4.3 ± 1.1	8.5 ± 3.0	9.7 ± 2.3 *	5.9 ± 1.7	4.1 ± 1.7	7.1 ± 1.6
Pancreatic juice trypsin output (U/h/kg b.wt.)						
Preprandial	1.1 ± 0.3	4.8 ± 1.7	4.6 ± 1.8 *	2.4 ± 1.1	1.3 ± 0.4	0.9 ± 0.7
Prandial	5.3 ± 1.1	11.0 ± 4.2	11.1 ± 3.7	5.3 ± 2.7	2.9 ± 1.1	3.4 ± 1.4
Postprandial	2.6 ± 0.7	6.7 ± 2.5	7.3 ± 2.2 *	3.1 ± 1.4	2.0 ± 0.7	2.8 ±1.5
Bile outflow (mL/h/kg b.wt.)						
Preprandial	2.9 ± 0.7	4.3 ± 0.7	4.4 ± 0.5 *	3.8 ± 0.3	4.1 ± 0.7	2.7 ± 0.4
Prandial	3.8 ± 0.7	3.9 ± 0.6	3.8 ± 0.3	3.0 ± 0.4	4.0 ± 0.7	2.9 ± 0.6
Postprandial	4.1 ± 0.6	3.2 ± 0.3	3.1 ± 0.5	2.6 ± 0.4 *	3.2 ± 0.6	2.5 ± 0.2 *

*—*p* < 0.05 differences between experimental and control treatments at the same secretory phase (Wilcoxon test).

**Table 8:** Pancreatic juice secretion of control (connected bile and PJ tubbing), bicarbonates infused instead of bile during the exp. and after overnight (13 h) bile diversion and bile or bile salts id. administration at various concentrations within 2 h (Mean values from *n* = 6 ± SEM).

Pancreatic Secretory Phase	Treatment						
	Control	B−, PJ+	B−, PJ+, (NaHCO_3_^−^)+	B−, PJ+, BAS (0.02 M)	B−, PJ+, BAS (0.04 M)	B−, PJ+, BAS (0.08 M)	B−, PJ+, B+
Pancreatic juice outflow (mL/h/kg b.wt.)							
Preprandial	1.0 ± 0.4	2.9 ± 0.8	3.5 ± 0.7 *	2.7 ± 0.5 *	3.3 ± 0.6	3.3 ± 0.7	1.5 ± 0.4
Prandial	3.5 ± 0.3	4.8 ± 0.6	4.8 ± 0.7	4.1 ± 0.6	3.2 ± 0.5	2.8 ± 1.0	3.5 ± 0.6
Postprandial 1st h	2.1 ± 0.1	4.6 ± 0.6 **	4.2 ± 0.6 *	2.3 ± 0.1	3.0 ± 0.5	2.4 ± 0.9	3.6 ± 0.8
Postprandial 2nd h	2.0 ± 0.3	4.3 ± 0.6 **	4.8 ± 0.5 ***	3.4 ± 0.5 *	2.6 ± 0.5	3.7 ± 1.3	3.2 ± 0.5 *
Postprandial 3rd h	2.4 ± 0.4	3.7 ± 0.5	6.6 ± 0.4 ***	3.8 ± 0.5 *	3.3 ± 0.4	3.6 ± 0.5	2.9 ± 0.6
Pancreatic juice protein outflow (mg/h/kg b.wt.)							
Preprandial	3.0 ± 1.0	6.9 ± 1.8	7.2 ± 1.0 *	4.8 ±1.5	5.6 ± 1.6	9.3 ± 1.5 ***	4.3 ± 1.0
Prandial	36.6 ± 15.1	52.7 ± 12.8	20.8 ± 7.4	24.1 ± 8.1	23.1 ± 7.1	11.4 ± 5.1	25.6 ± 6.5
Postprandial 1st h	11.1 ± 2.0	28.2 ± 5.5 *	13.3 ± 2.8	14.3 ± 1.37	11.9 ± 2.9	9.3 ± 3.4	22.7 ± 6.8
Postprandial 2nd h	10.4 ± 2.7	23.8 ± 4.3 *	10.5 ± 0.8	14.9 ± 1.73	13.1 ± 3.1	11.5 ± 5.2	20.6 ± 5.4
Postprandial 3rd h	11.9 ± 3.1	26.7 ± 4.3 **	13.6 ± 1.5	15.2 ± 2.06	13.1 ± 3.3	11.2 ± 2.3	21.0 ±5.9
Pancreatic juice trypsin outflow (U/h/kg b.wt.)							
Preprandial	2.2 ± 0.9	45.5 ± 1.4	4.5 ± 1.5	3.7 ± 2.1	7.3 ± 2.1 *	6.7 ± 1.4 *	2.9 ± 0.8
Prandial	12.7 ± 3.0	39.7 ± 10.2 *	14.2 ± 6.8	16.5 ± 5.8	12.3 ± 4.4	9.1 ± 4.3	22.3 ±8.3
Postprandial 1st h	9.0 ± 2.0	23.0 ± 9.1 *	10.3 ± 4.0	9.7 ± 3.3	8.7 ± 3.0	7.8 ± 3.5	17.7 ± 5.6
Postprandial 2nd h	8.1 ± 2.3	18.7 ± 3.6 *	6.4 ± 1.9	11.4 ± 4.0	8.8 ± 2.7	9.3 ±5.0	16.1 ± 4.5
Postprandial 3rd h	8.5 ± 2.6	19.3 ± 2.5 *	7.6 ± 2.4	10.5 ± 3.6	9.6 ± 3.0	9.1 ± 2.7	13.9 ± 4.4
Bile outflow (mL/h/kg b.wt.)							
Preprandial	2.5 ± 0.3	2.1 ± 0.4	5.0 ± 2.0	3.6 ± 0.8	3.1 ± 0.5 *	2.5 ± 0.7	1.6 ± 0.1 *
Prandial	3.7 ± 0.4	1.4 ± 0.1 **	5.0 ± 1.2	2.3 ± 0.3 *	3.1 ± 0.3 *	2.5 ± 0.7	1.9 ± 0.1 *
Postprandial 1st h	3.7 ± 0.5	1.9 ± 0.3 *	3.7 ± 1.2	2.6 ± 0.3 *	4.0 ± 0.5	3.8 ± 0.7	3.3 ± 0.4
Postprandial 2nd h	3.0 ± 0.6	2.2 ± 0.4	3.3 ± 0.7	3.2 ± 0.4	3.1 ± 0.3	4.5 ± 1.3	3.7 ± 0.3
Postprandial 3rd h	3.3 ± 0.5	1.6 ± 0.3 *	4.1 ± 1.0	2.9 ± 0.3	3.9 ± 0.8	5.0 ± 0.6	2.5 ± 0.3

* = *p* < 0.05, ** = *p* < 0.01, *** = *p* < 0.001 differences between treatments at the same secretory phase (Wilcoxon test).

**Table 9:** Leptin secretion in bile (ng/kg body mass/hour) under conditions of preserved and interrupted bile flow to the duodenum in piglets (Mean values from *n* = 6 ± SE).

Items	Piglets with Preserved PJ and Bile Flow to Duodenum	Piglets with Interrupted Bile Flow to Duodenum
Before feed intake	40.6 ± 12.2	33.0 ± 6.6
Feeding phase	75.4 ± 24.7 ^a^	15.3 ± 5.7 *^b^
Post-feeding phase (1 h)	69.5 ± 17.5 ^a^	23.0 ± 10.4 ^b^
Post-feeding phase (2 h)	62.4 ± 5.5 ^a^	20.0 ± 7.4 ^b^
Post-feeding phase (3 h)	58.0 ± 21.9	19.6 ± 8.7

^a,b^ = *p* < 0.05. Different letters indicate statistically significant differences between treatments (Wilcoxon test). * = *p* < 0.05 indicates significant difference from the pre-feeding sample.

**Table 10:** Summary of multilevel feedback regulation of pancreatic exocrine secretion in piglets.

Level	Signal	Core Observation	Interpretation
I	Pancreatic proteases	PJ diversion ↑ secretion 2–3×; pancreatin restores inhibition	Primary inhibitory feedback
II	Distal protease sensing	Ileal pancreatin suppresses secretion	Feedback beyond duodenum
III	CCK/secretin pathways	Tarazepide ↓ ~54%; combined blockade ↓ ~65%	Parallel effector pathways
IV	Bile acids/leptin	Bile diversion ↑ secretion; bile acids modulate; leptin ↓	Phase-dependent modulation

↑ indicates an increase in pancreatic exocrine secretion, whereas ↓ indicates a decrease or inhibition of secretion.

## Data Availability

The data supporting the findings of this study are available from the corresponding author upon reasonable request. Public access to the data is restricted due to ethical approval conditions and institutional policies regarding animal experimentation.

## Abbreviations

The following abbreviations are used in this manuscript:

ATP	Adenosine Triphosphate
BAPNA	Nα-benzoyl-DL-arginine p-nitroanilide
BSA	Bovine Serum Albumin
cAMP	Cyclic Adenosine Monophosphate
CCK	Cholecystokinin
CCK-33	CCK Octapeptide
CCK1	Peripheral Cholecystokinin Receptor (CCK-A; A—Alimentary)
CCK2	Central Cholecystokinin Receptor (CCK-B; B—Brain)
CGRP	Calcitonin Gene-Related Peptide
EDTA	Ethylenediaminetetraacetic Acid
GIP	Gastric Inhibitory Peptide
GLP 1–36	Glucagon-Like Peptide 1–36 (Glucagon-like Growth Factor I)
GLP2	Glucagon-Like Peptide 2 (Glucagon-like Growth Factor II)
GRP	Gastrin-Releasing Peptide
H+/K+-ATPase	Proton-potassium pump adenosine triphosphatase
id	Intraduodenal
IgG	Immunoglobulin G
il	Intraileal
K_3_EDTA	Tripotassium ethylenediaminetetraacetate
iv	Intravenous
I-cells	CCK-producing cells
b.w.	Body weight
MMC	Migrating Motor Complex
MP	Monitor Peptide
mRNA	messenger Ribonucleic Acid
n	Number of animals in the experiment
PP	Pancreatic Polypeptide
PYY	Pepetide YY
PYY 1–36	Peptide YY fragment 1–36
PYY 3–36	Peptide YY fragment 3–36
SE	Standard Error
SRP	Secretin-Releasing Peptides
VIP	Vasoactive Intestinal Peptide
PJ	Pancreatic Juice
PEG	Pegylated Leptin
LCRF	Luminal CCK-Releasing Factor
PLC	Phospholipase C
NTS	Nucleus Tractus Solitarius
AC6	Adenylyl Cyclase 6
SCTR	Secretin Receptors
PKA	Protein Kinase A
PKC	Protein Kinase C
IP_3_R	Inositol 1,4,5-triphosphate Receptor
FXR	Farnesoid X Receptor
NR1H4	Nuclear Receptor Subfamily 1 Group H Member 4
CDCA	Chenodeoxycholic Acid
FGF15/19	Fibroblast Growth Factor 15/19
CYP7A1	Cholesterol 7α-hydroxylase
TGR5	Takeda G Protein-Coupled Receptor 5 (GPBAR1)
POMC	Pro-opiomelanocortin
B	Bile
U	Unit

## Appendix A

### Appendix A.1

**Table A1:** Scheme of experiments 1–14 performed using experimental model I.

Experimental Options	Experiment Number													
	1	2	3	4	5	6	7	8	9	10	11	12	13	14
Pancreatic juice infusions into duodenum	+	+	+	+	-	-	-	-	+	+	+	-	-	-
Pancreatin infusions into duodenum	-	20%	100%	200%	-	20%	100%	200%	-	-	-	-	-	-
Pancreatin infusions into ileum	-	-	-	-	-	-	-	-	20%	100%	200%	20%	100%	200%

Legend: + administration of pancreatic juice to the duodenum in amounts equal to those secreted under control into the duodenum. - no administration of pancreatic juice or pancreatin to the duodenum or to the ileum. 20% infusion of pancreatin solution with trypsin activity corresponding to 20% of trypsin activity during the feeding period. 100% infusion of pancreatin solution with trypsin activity corresponding to trypsin activity during the feeding period. 200% infusion of pancreatin solution with trypsin activity corresponding to double the trypsin activity during the feeding period.

**Table A2:** Scheme of experiments 15–21 performed using model II with administration of omeprazole, tarazepide, and bicarbonates.

Experiment Number	Experiment Options				
	Pancreatic Juice	Bile	Omeprazole	Tarazepide	Bicarbonates *
15	+	+	-	-	-
16	+	-	-	-	-
17	-	-	-	-	-
18	+	+	+	-	-
19	+	+	-	+	-
20	+	+	+	+	-
21	+	-	-	-	+

Legend: + Administration of pancreatic juice, bile, bicarbonate, omeprazole, or tarazepide during the experiment. - No administration of pancreatic juice, bile, bicarbonate, omeprazole, or tarazepide during the experiment. * Bicarbonate was administered to the duodenum in amounts corresponding to the volume of bile secreted in the 30 min period of bile collection immediately prior to its administration.

**Table A3:** Scheme of experiments 22–26 performed using model II with preserved return of bile and pancreatic juice to the duodenum and with interruption of their flow to the duodenum, as well as with infusions of bile acid salt solutions to the duodenum.

Experiment Number	Experiment Options			
	Pancreatic Juice	Bile During 10 h Before Experiments	Bile During Experiments	Bile Acids Salts Solutions
22	+	+	+	-
23	+	-	-	-
24	+	-	-	0.02 M
25	+	-	-	0.04 M
26	+	-	-	0.08 M

Legend: + Administration of pancreatic juice or bile to the duodenum before or during the experiment. - No administration of bile to the duodenum before or during the experiment. 0.02 M; 0.04 M; 0.08 M Molarity of bile acid salt solutions administered to the duodenum during the feeding phase and in the first hour of the post-feeding phase.
